# Supplementary figures and images for: Assessment of Augmented Reality Glasses for Spatial Tracking and Intraoperative Annotation in Veterinary Surgery
Source: bioRxiv. 2025 Dec 29:2025.12.18.695281. Preprint. [Version 1] doi: 10.64898/2025.12.18.695281 (PMC12773008; doi:10.64898/2025.12.18.695281)

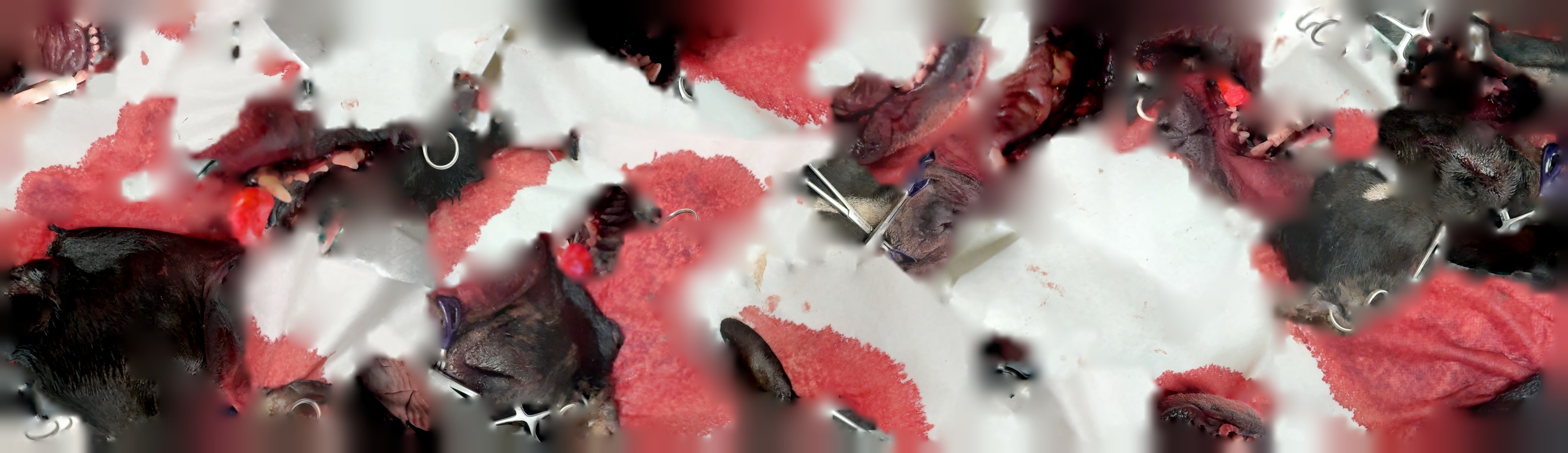

Supplement: Supplement 3 [file media-3.zip › Dog_Scan/Dog_Scan.jpg]
